# Supplementary material for: Exploring Gut Microbiome in Predicting the Efficacy of Immunotherapy in Non-Small Cell Lung Cancer
Source: Cancers (Basel). 2022 Nov 2;14(21):5401. doi: 10.3390/cancers14215401 (PMC9656313; doi:10.3390/cancers14215401)
Supplement: Supplementary file 1 [file cancers-14-05401-s001.zip › Supplemental Materials.pdf]

Method

(1) Data set and metadata collection

We have two datasets (DS1 and DS2) in this study. The details of DS1 is shown below in Patients and samples. For DS2, we began by performing literature searches on the SRA database in NCBI for any publications related to NSCLC immunotherapy with published data of the gut microbiome by "(NSCLC gut) AND bioproject_sra[filter] NOT biopro-ject_gap[filter]" with cutoff date of Dec. 14, 2021. This resulted 5 records. 2 of the 5 records contain whole metagenome shotgun sequencing (WGS) data and only one[1] has metadata associated with WGS data, which was referred as DS2 in this paper. Broadening the search criteria to lung cancer resulted in 16 records but no further WGS data (WGS presented with metadata) were found to be usable.

(2) Patients and samples (dataset name)

14 NSCLC patients who received immunotherapy and have known PFS were selected from our previous study [2] (referred as DS1, can be accessed from PRJNA866654). Their pretreatment baseline fecal samples were collected and extracted DNA sequenced on Illumina HiSeq for 2 × 150pb; NexteraXT preparation was used, by COSMOSID®. DS2 contained 65 additional pretreatment samples. For PFS study, 6 patients with long PFS and 3 patients with short PFS were included in DS1, and 7 samples with long PFS and 34 samples with short PFS were included in DS2. We omitted patients with PFS between 3 and 6 months to further contrast the gut microbiome of patients with long vs. short PFS. We later applied our trained models to predict treatment response. We grouped patients with complete and partial response as responder (R), whereas those with stable disease, progression, and death as non-responder (NR). With this design, all 79 patients (14 from DS1 plus

65 from DS2) were included for analysis, with 8 R and 6 NR in DS1, and 12 R and 53 NR in DS2. RECIST 1.1 criteria [3] was used to assess the treatment response.

(3) Quality control

All raw metagenomic data were quality controlled by Trimmomatic[4] (v0.38). For DS1, the raw reads were pair-ended, and the following script was used:

```
java -jar trimmomatic-0.38.jar \
PE read1.fastq read2.fastq \
forward.paired.fq.gz forward.unpaired.fq.gz \
reverse.paired.fq.gz reverse.unpaired.fq.gz \
ILLUMINACLIP: adapters.fa:2:30:10 \
LEADING:30 \
TRAILING:30 \
SLIDINGWINDOW:4:15 \
HEADCROP:20 \
MINLEN:35 -threads 16;
```

Where,

- ILLUMINACLIP: adapters.fa:2:30:10 specified the adapter library.
- *LEADING:30* specified the minimum quality required to keep a base from the beginning.
- *TRAILING:30* specified the minimum quality required to keep a base from the end.
- *SLIDINGWINDOW:4:15* specified the window size to be 4 bases and required the average quality to be 15.

- *HEADCROP:20* specified to discard 20 bases from the start of the read.
- *MINLEN:35* specified the minimal length of reads to be kept as 35 bases.
- *-threads 16* assigned 16 CPUs to Trimmomatic.

For DS2, the raw reads were single-ended, and the following script was used:

```
java -jar trimmomatic-0.38.jar \
SE reads.fastq reads.trimed.fastq \
ILLUMINACLIP:TruSeq3-SE.fa:2:30:10 \
LEADING:30 \
TRAILING:30 \
SLIDINGWINDOW:4:15 \
CROP:280 \
MINLEN:35 -threads 16;
```

Where,

- ILLUMINACLIP:TruSeq3-SE.fa:2:30:10 specified the adapter library.
- *LEADING:30* specified the minimum quality required to keep a base from the beginning.
- *TRAILING:30* specified the minimum quality required to keep a base from the end.
- *SLIDINGWINDOW:4:15* specified the window size to be 4 bases and required the average quality to be 15.
- *CROP:280* specified to cut the read to 280 bases by removing bases from the end.
- *MINLEN:35* specified the minimal length of reads to be kept as 35 bases.
- *-threads 16* assigned 16 CPUs to Trimmomatic.

The per-base quality content plots of both datasets were shown in Figure S1.

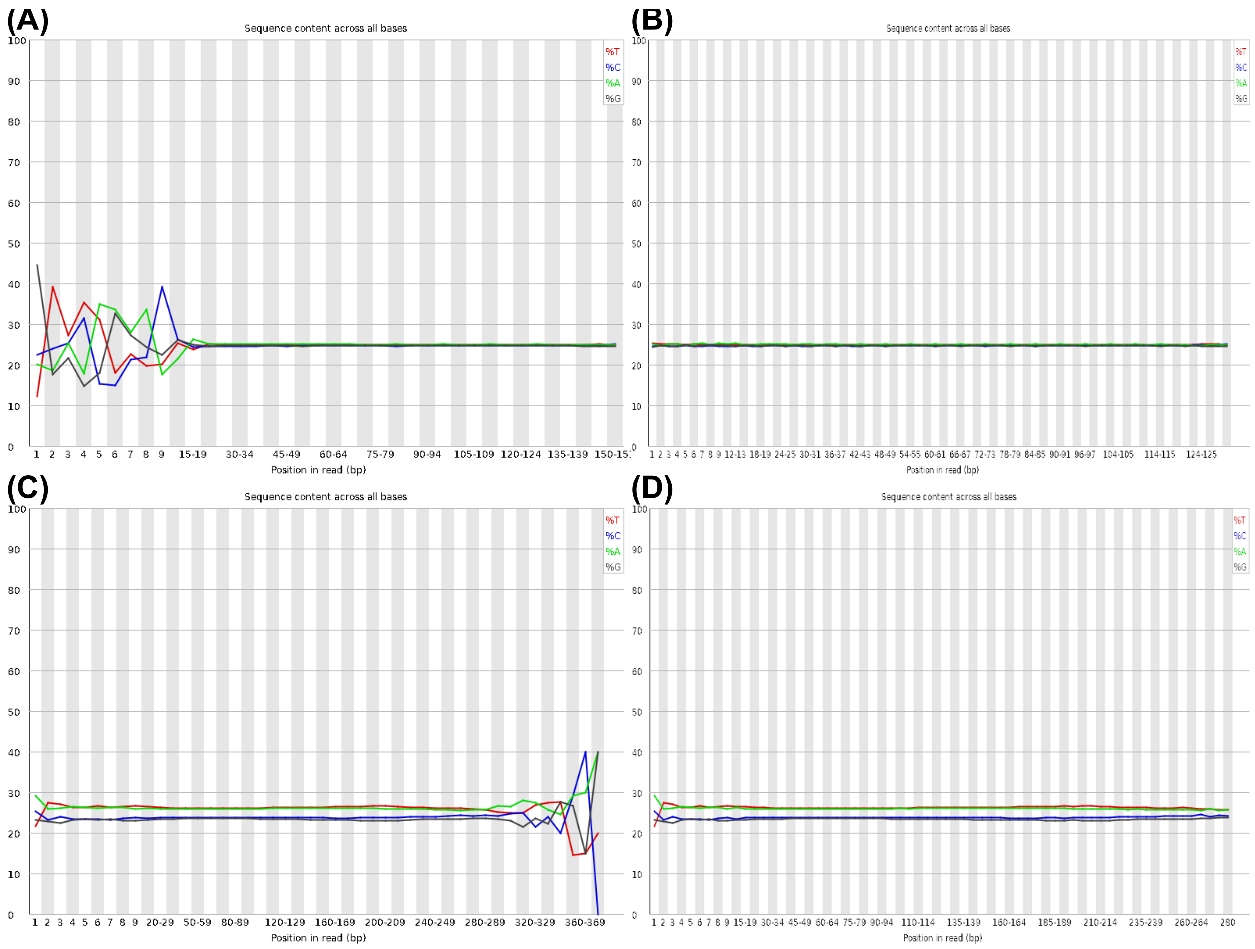

Figure S1. Examples illustrating per-base content before and after quality control. (A) Per-base content of forward reads of samples from patient JZLC 04 before quality control. (B) Per-base content of forward reads of samples from patient JZLC 04 after quality control. (C) Per-base content of sample ERR2213660 before quality control. (D) Per-base content of sample ERR2213660 after quality control.

(4) Removing human genome sequence contamination

BWA [5] (0.7.16a-r1185-dirty) was used to map the reads after quality control to human genome (hg38) with default parameters:

```
bwa index hg38.fa

bwa -t 16 hg38.fa reads.trimed.fastq > map.sam
```

The first line was to index the human genome and it only needs to be executed once.

After getting the mapping results in the SAM file, we are going to remove all reads (DS2) or read pairs (DS1) if the read was reported to be aligned to the human genome. The mapping rate, i.e., percentage of reads were removed after this step was listed in Supplemental Table 7.

(5) Taxonomy annotation

MetaPhlAn[6] (v3.0.6) was used to annotate the composition of microbial communities against its own database, mpa_v30_CHOCOPhlAn_201901, containing marker genes from about 99,500 bacterial reference genomes and about 500 eukaryotic reference genomes.

For DS1 and DS2, the following script was used: (all parameters are the same except for input and output filename)

```
metaphlan forward.paired.fq.gz reverse.paired.fq.gz \
-t rel_ab_w_read_stats \
--nproc 16 --input_type fastq \
--bowtie2out bowtie2.bz2 -o OTU.tsv
```

Where,

- *-t rel_ab_w_read_stats* specified MetaPhlAn to profile metagenomes in terms of relative abundances and estimate the number of reads coming from each clade (raw read count).
- *--input_type fastq* informed the input data was in fastq format.
- *--nproc 16* assigned 16 CPUs to MetaPhlAn.
- *--bowtie2out bowtie2.bz2 -o OTU.tsv* specified the filename of MetaPhlAn output: *bowtie2.bz2* is the file for saving the output of BowTie2 and *OTU.tsv* is the tab-separated output file of the predicted taxon abundances.

(6) Protein sequence annotation

The UProC[7] (v1.2.0) was used to analyze the protein sequence against databases created from Pfam[8] (28.0) and KEGG Orthology (released March 2014), respectively.

For analyzing DS1 against Pfam database, the following script was used:

```
uproc-dna -t 16 -o pfam.csv \
/path/to/uproc/pfam \
/path/to/uproc/model \
forward.paired.fq.gz reverse.paired.fq.gz
```

Where,

- *-t 16* assigned 16 CPUs to UProC.
- *-o pfam.csv* specified the comma-separated output file of the predicted protein family abundances.
- */path/to/uproc/pfam* informed the directory to Pfam database, which can be downloaded from the UProC website.
- */path/to/uproc/model* informed the directory to UProC prediction model, which can be downloaded from the UProC website.
- forward.paired.fq.gz reverse.paired.fq. specified the input filename.

To analyze DS1 against the KEGG Orthology database, we changed */path/to/uproc/pfam* to */path/to/uproc/kegg*, the directory to KEGG Orthology database. To analyze DS2, we changed the *forward.paired.fq.gz reverse.paired.fq.gz* to *reads.trimed.fastq*, the filename of DS2.

(7) Microbiome community study

The characteristics of the microbiome community were studied using web-based tool MicrobiomeAnalyst[9], which included alpha-diversity, beta-diversity, and comparative analysis to assess the differential abundances separating the microbiomes (metagenomeSeq[10]).

(8) Hierarchical clustering

The unsupervised clustering analysis was performed using *clustermap* provided by seaborn[11] (v0.11.0). The ranking of each taxa was determined by metagenomeSeq from MicrobiomeAnalyst. For protein sequence annotation, the ranking of protein family (for Pfam) or KEGG Orthology was determined by DESeq2[12] (v1.22.2). The raw read count was normalized to reads per kilobase million (RPKM) before generating the heatmap, which used the per-feature (taxonomy, protein family, or KEGG Orthology) Z-score for hierarchical clustering.

(9) Prediction

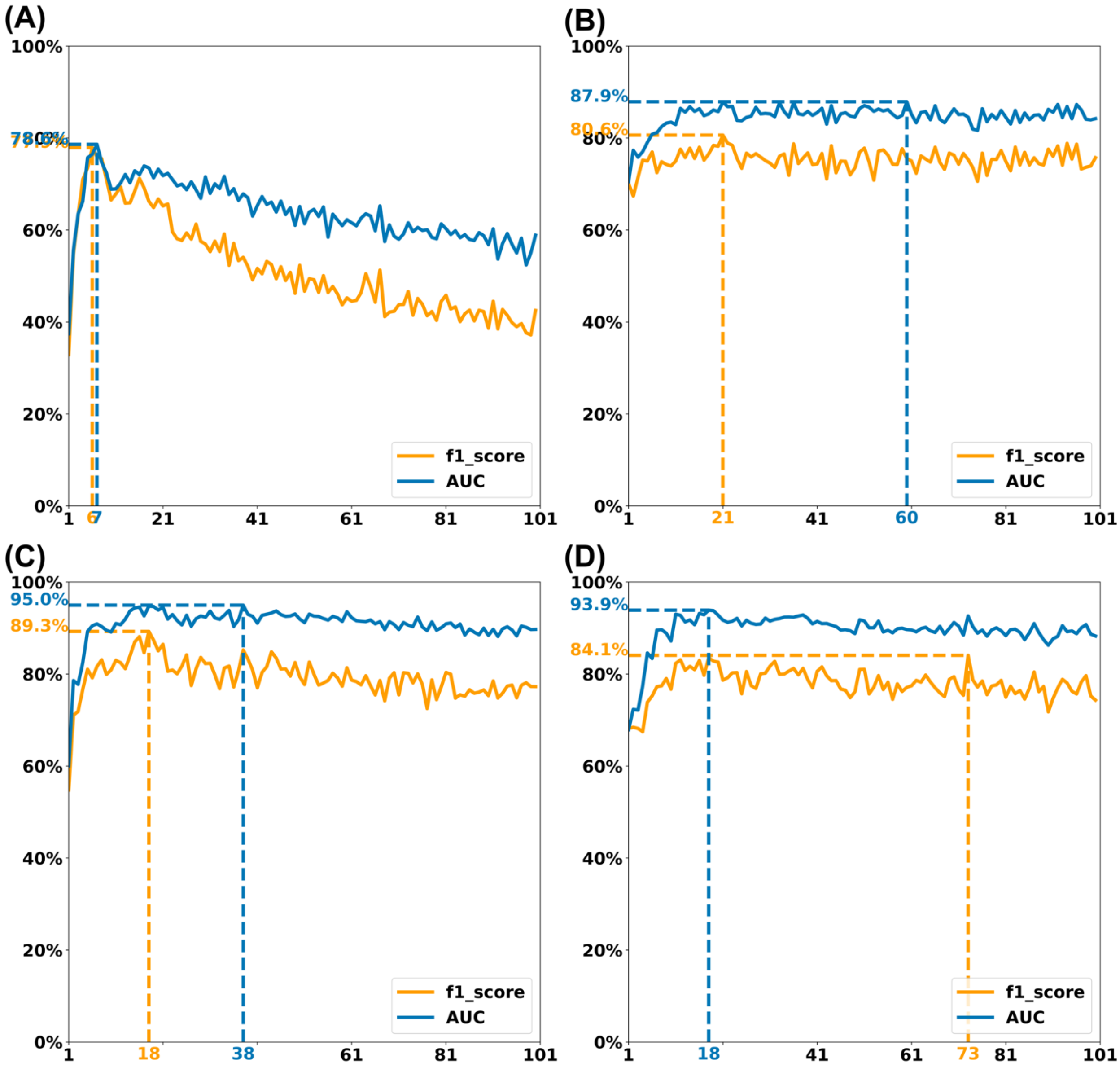

Figure S2. The fine-tuning processes. (A) the fine-tuning process of taxonomic profile, the x-axis indicates number of ranked features used in prediction, the y-axis indicates the performance score. (B) the fine-tuning process of KEGG Orthology profile. (C) the fine-tuning process of Pfam profile. (D) the fine-tuning process of the combined taxonomic profile, Pfam and KEGG Orthology.

Random forest (RF) classifier and multilayer perceptron (MLP) classifier from scikit-learn [13] (v0.24.2) was used to predict if the patient had long PFS or was in the R group. When predicting long PFS, the training-testing splitting must satisfy the following: (a) the training dataset contains 35 samples, and the testing dataset contains 15 samples; (b) the testing dataset must contain 4 samples with long PFS. When training the model, we first trained the model using all available features.

Noticing that the number of features is larger than the number of samples, we calibrated the changing of the prediction score with respect to the number of features, which is ranked by Gini importance (defined as the normalized total reduction of the Gini impurity brought by that feature). We selected the top feature sets which maximized the AUC score: the numbers of features were 7, 60, 38, and 18 for taxonomical, KEGG Orthology, Pfam and the combination of all profiles, respectively (shown in Figure S2). The early stopping was enabled for MLP to prevent overfitting to training set; all other parameters were left default.

To measure performance, the true positive (TP) was defined as the subset of patients with PFS above 6 months who were predicted to have long PFS and the true negative (TN) was defined as the subset of patients with PFS below 3 months who were predicted to have short PFS. Similarly, false positives (FP) were defined as the subset of patients with PFS below 3 months who were predicted to have long PFS, and false negatives (FN) were defined as the subset of patients with PFS above 6 months who were predicted to have short PFS. The performance of our model was measured by sensitivity, precision, F-score, and accuracy:

$$\text{sensitivity} = \frac{TP}{TP + FN}$$

$$\text{precision} = \frac{TP}{TP + FP}$$

$$\text{F} - \text{score} = \frac{2 \cdot \text{sensitivity} \cdot \text{precision}}{\text{sensitivity} + \text{precision}}$$

$$\text{accuracy} = \frac{TN}{TN + FN}$$

To validate our findings, the same procedure was repeated 100 times and averages of 4 performance matrices were computed using different stringency cutoffs to generate the receiver operating characteristic curve (ROC). The ROC curves were extrapolated to the points (sensitivity: 0, precision: 1) and (sensitivity: 1, precision: 0) to calculate the AUC score.

We further studied the predictive power of our Pfam feature set by directly applying the RF and MLP model in order to classify patients into R/NR groups. In this case, the 50 samples used in the PFS study were used as the training set (with PFS labels). The testing set included the 79 samples with R/NR group label. Stochastic predictor was used as benchmark and the exact distribution of its performance was approximated by a 1000-iteration simulation.

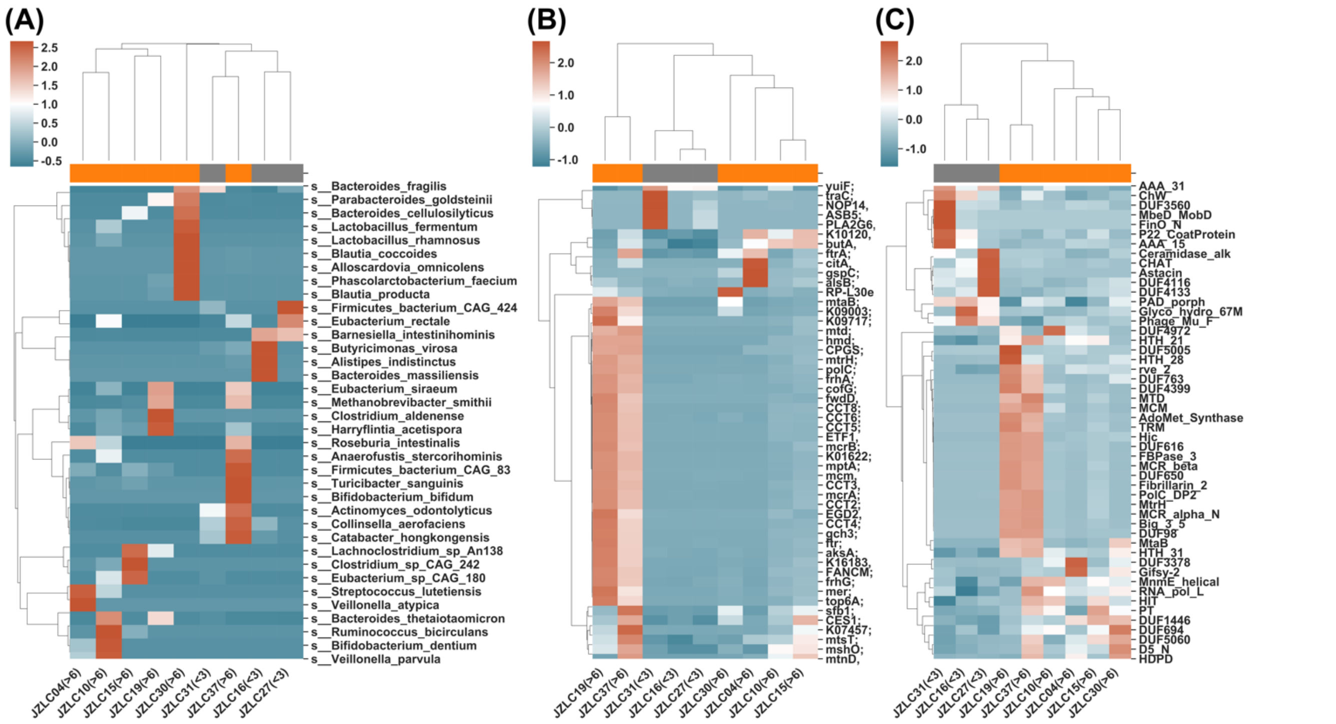

Figure S3. The hierarchical clustering of DS1 of 9 sample. (A) The hierarchical clustering of z-score of abundances of most differential taxa (36) between PFS Long (orange) and PFS Short samples (grey). (B) The hierarchical clustering of top 50 differential microbiota as per KEGG Orthology. (C) The hierarchical clustering of the top 50 differential protein families.

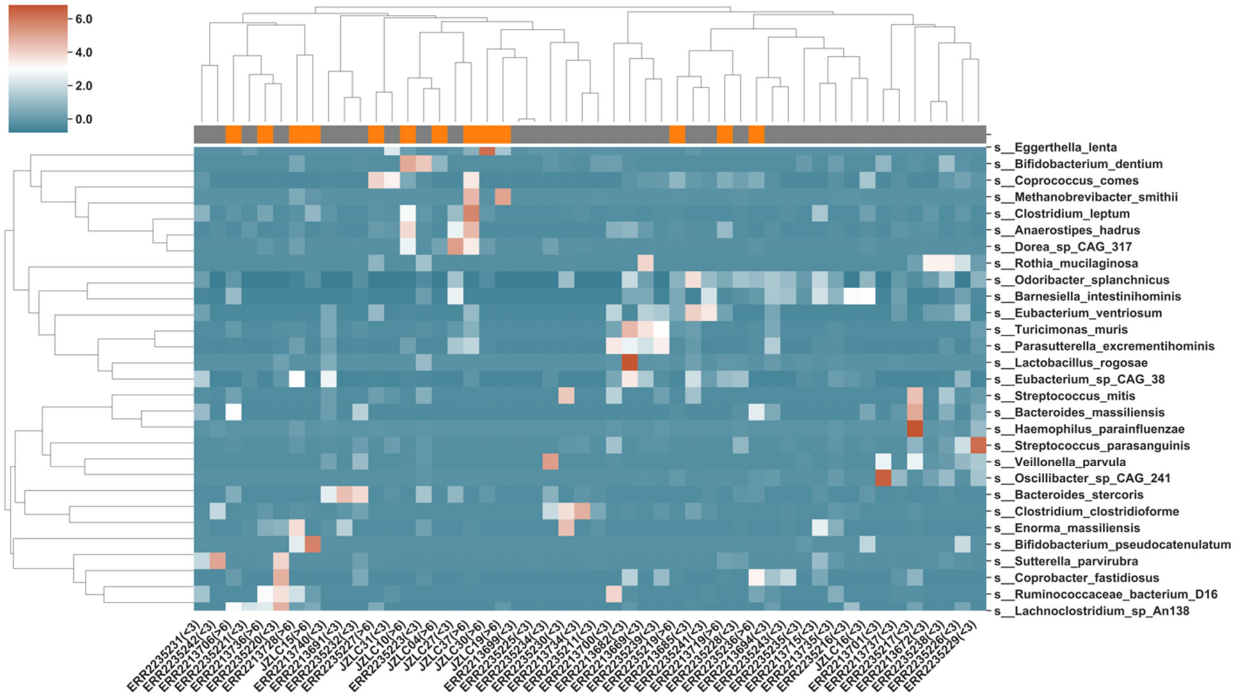

Figure S4. The hierarchical clustering of z-score of abundances of most differential taxa (29) between PFS Long (orange) and PFS Short (grey) on DS1 and DS2 together.

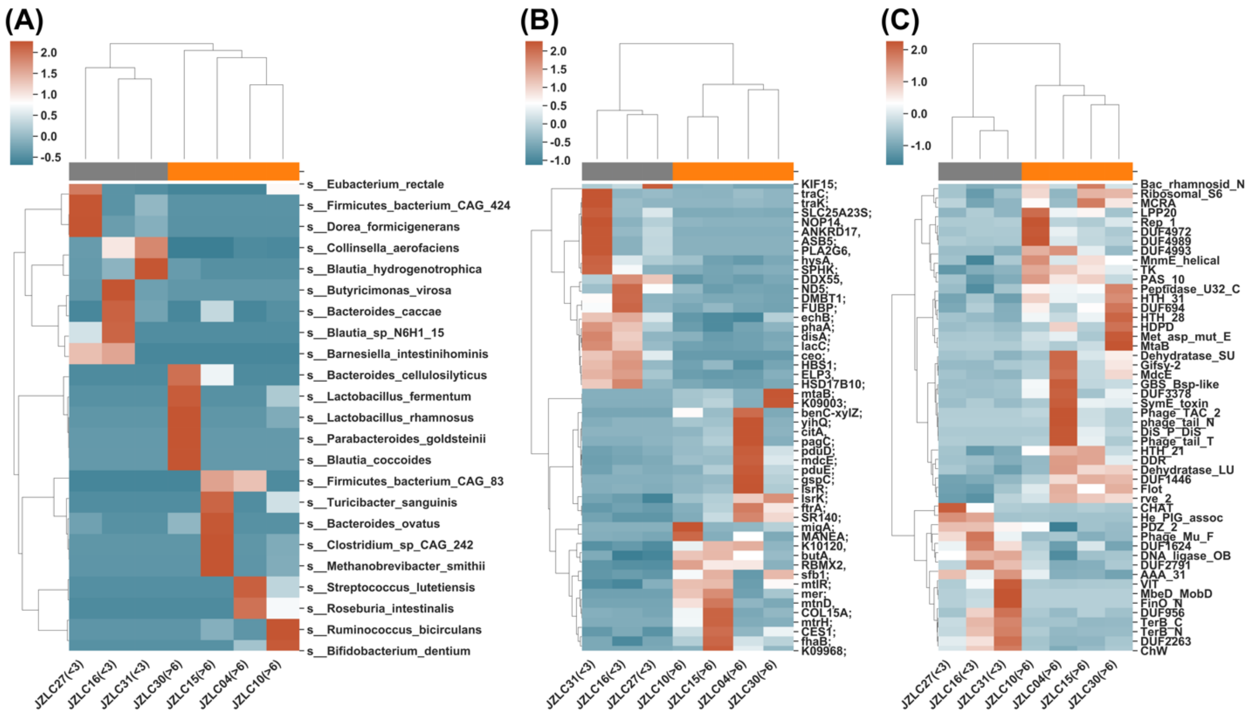

Figure S5. The hierarchical clustering of 7 sample on DS1. (A) The hierarchical clustering of z-score of abundances of most differential taxa (23) between PFS Long and PFS Short samples, i.e., Figure 2 (C) in main manuscript. (B) The hierarchical clustering of top 50 differential microbiota as per KEGG Orthology, i.e., Figure 3(A) in main manuscript. (C) The hierarchical clustering of the top 50 differential protein families, i.e., Figure 3(C) in main manuscript.

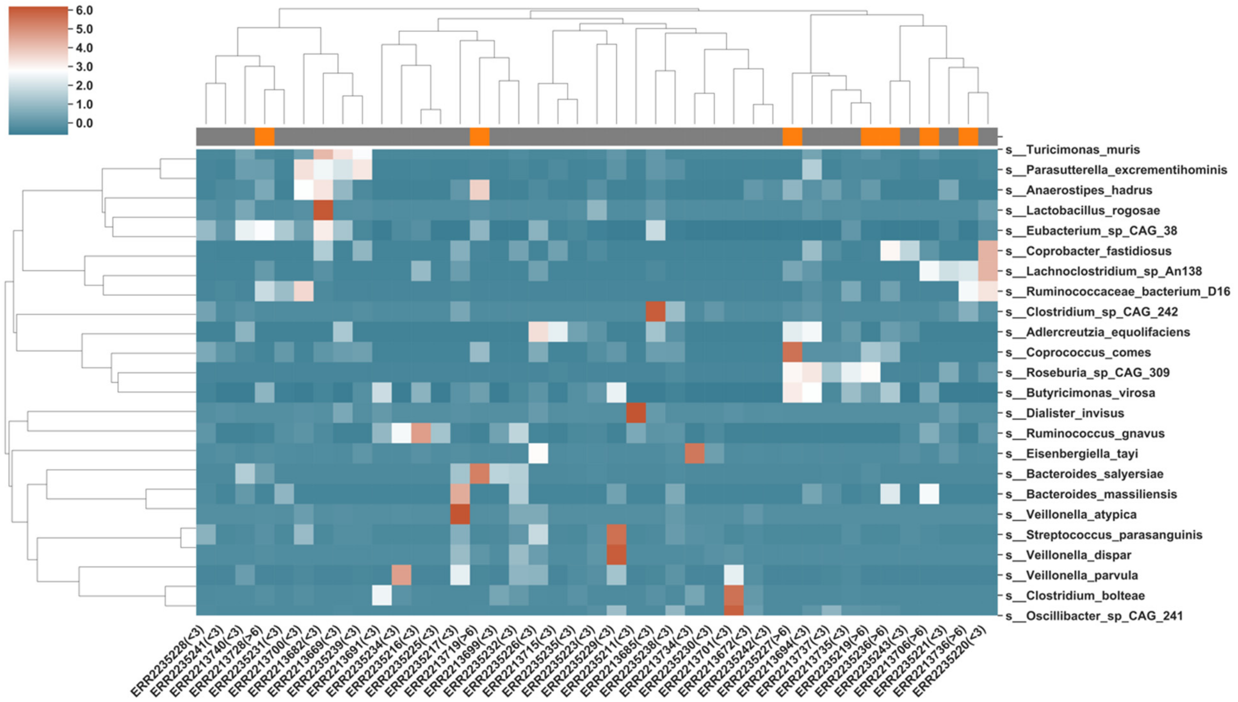

Figure S6. The hierarchical clustering of z-score of abundances of most differential taxa (24) between PFS Long and PFS Short samples on DS2, i.e., Figure 2 (D) in main manuscript.

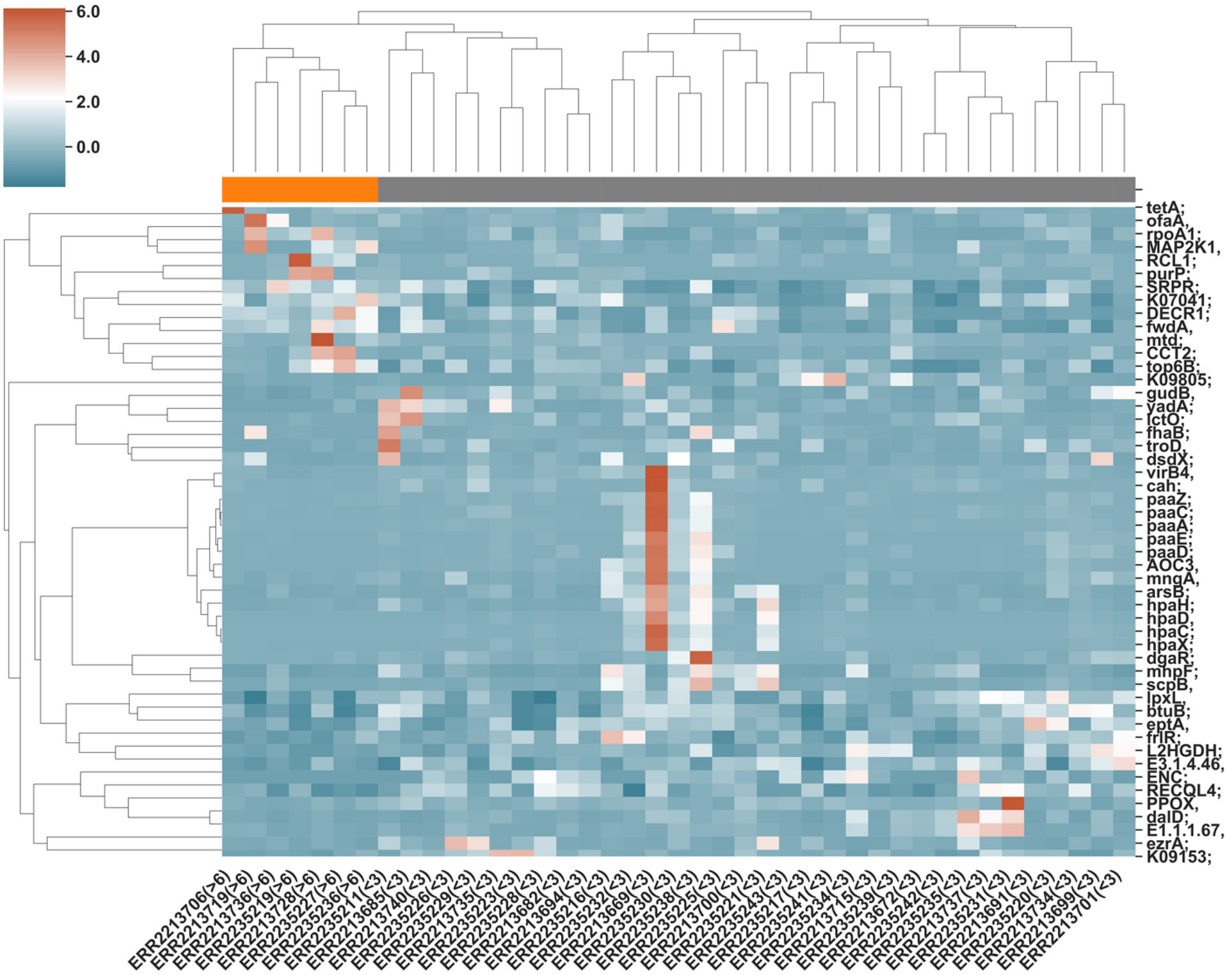

Figure S7. The hierarchical clustering of z-score of abundances of 50 most differential protein families using KEGG Orthology profile on DS2, i.e., Figure 3 (B) in main manuscript.

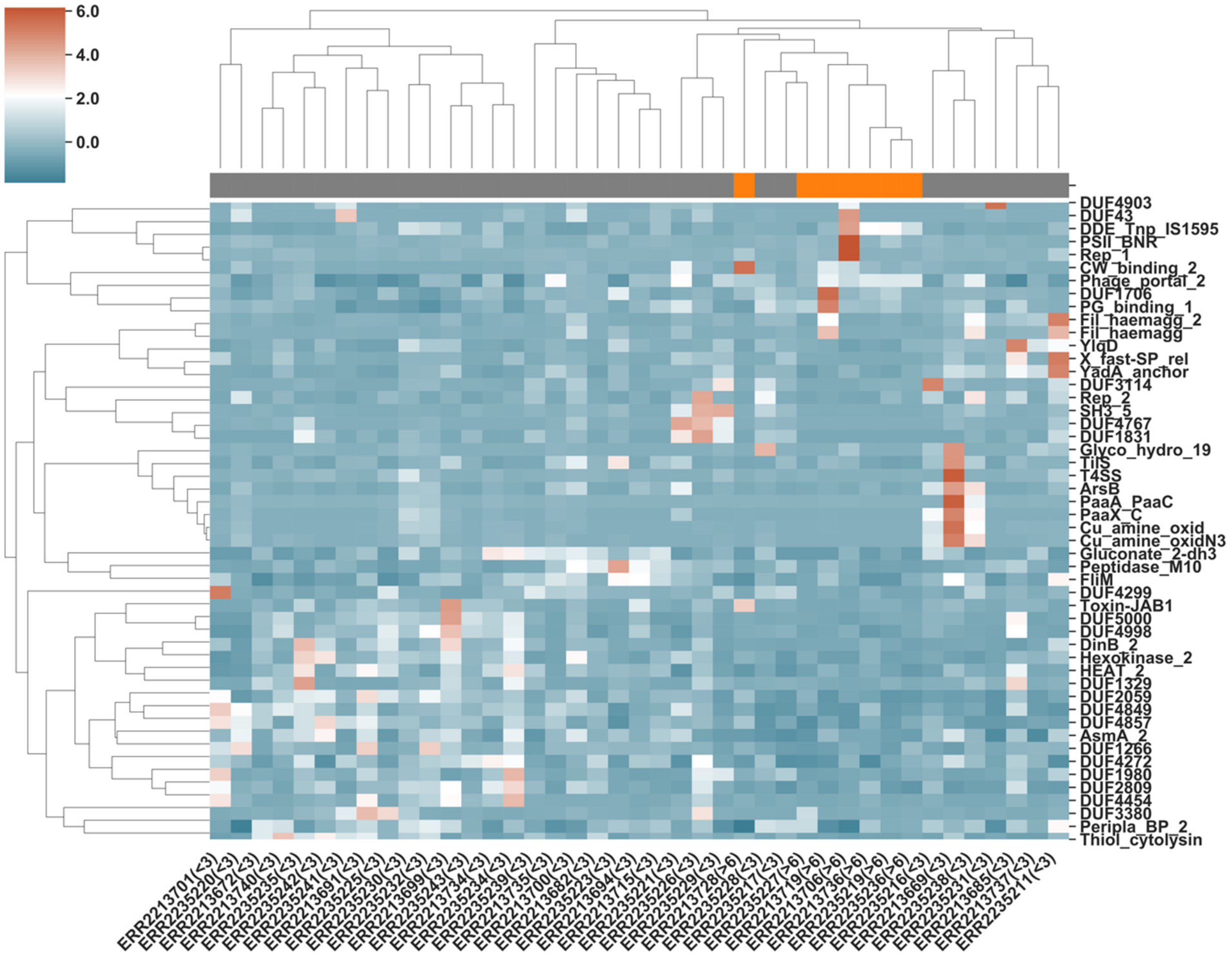

Figure S8. The hierarchical clustering of z-score of abundances of 50 most differential protein families using Pfam profile on DS2, i.e., Figure 3 (D) in main manuscript.

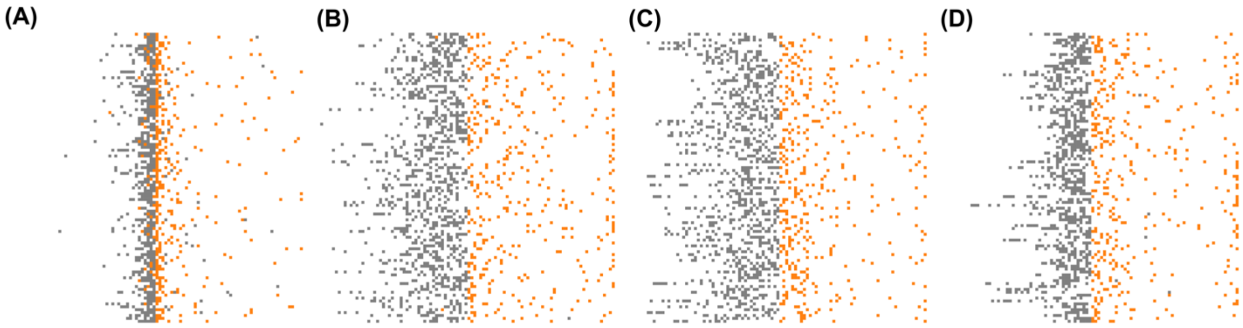

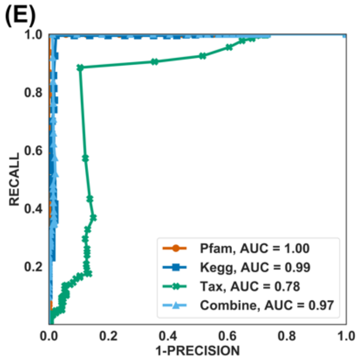

	Sensitivity	Precision	F1-score	Accuracy
Tax	88.5	89.5	87.7	96.1
KEGG	99.5	97.6	98.4	99.8
Pfam	**100**	**99.8**	**99.9**	**100**
Combine	**100**	98.8	99.3	**100**

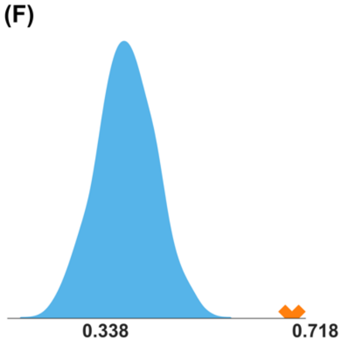

Figure S9. Prediction of MLP classifier. (A-D) Prediction score distribution using taxonomic profile, the KEGG Orthology profile, Pfam profile and combined profiles, respectively. Each row represents one random testing experiment, 100 experiments in total. Orange dots indicate PFS Long samples;

grey dots indicate PFS Short samples. (E) Performance of prediction. The left panel shows the averaged ROC curve of 4 profiles; the right panel shows the averaged prediction score using the default (0.5) prediction score cutoff. (F) Using the MLP and Pfam feature set from PFS study to predict responders, measured by F1-score. The light blue curve is the exact distribution of random guessing, and the orange cross is the actual performance of the trained model.

Reference

1. Routy, B., et al., *Gut microbiome influences efficacy of PD-1–based immunotherapy against epithelial tumors.* Science, 2018. **359**(6371): p. 91-97.
2. Chau, J., et al., *Prospective correlation between the patient microbiome with response to and development of immune-mediated adverse effects to immunotherapy in lung cancer.* BMC Cancer, 2021. **21**(1): p. 808.
3. Schwartz, L.H., et al., *RECIST 1.1 - Standardisation and disease-specific adaptations: Perspectives from the RECIST Working Group.* European journal of cancer (Oxford, England : 1990), 2016. **62**: p. 138-145.
4. Bolger, A.M., M. Lohse, and B. Usadel, *Trimmomatic: a flexible trimmer for Illumina sequence data.* Bioinformatics (Oxford, England), 2014. **30**(15): p. 2114-2120.
5. Li, H. and R. Durbin, *Fast and accurate short read alignment with Burrows–Wheeler transform.* bioinformatics, 2009. **25**(14): p. 1754-1760.
6. Beghini, F., et al., *Integrating taxonomic, functional, and strain-level profiling of diverse microbial communities with bioBakery 3.* eLife, 2021. **10**: p. e65088.
7. Meinicke, P., *UProC: tools for ultra-fast protein domain classification.* Bioinformatics, 2014. **31**(9): p. 1382-1388.
8. Mistry, J., et al., *Pfam: The protein families database in 2021.* Nucleic Acids Research, 2020. **49**(D1): p. D412-D419.
9. Chong, J., et al., *Using MicrobiomeAnalyst for comprehensive statistical, functional, and meta-analysis of microbiome data.* Nature Protocols, 2020. **15**(3): p. 799-821.
10. Paulson, J.N., et al., *Differential abundance analysis for microbial marker-gene surveys.* Nature Methods, 2013. **10**(12): p. 1200-1202.
11. Waskom, M.L., *Seaborn: statistical data visualization.* Journal of Open Source Software, 2021. **6**(60): p. 3021.
12. Love, M.I., W. Huber, and S. Anders, *Moderated estimation of fold change and dispersion for RNA-seq data with DESeq2.* Genome Biology, 2014. **15**(12): p. 550.
13. Pedregosa, F., et al., *Scikit-learn: Machine Learning in Python.* J. Mach. Learn. Res., 2011. **12**(null): p. 2825–2830.
